# Proactive expert system intervention to prevent or quit at-risk alcohol use (PRINT): study protocol of a randomized controlled trial

**DOI:** 10.1186/s12889-018-5774-1

**Published:** 2018-07-09

**Authors:** Sophie Baumann, Andreas Staudt, Jennis Freyer-Adam, Ulrich John

**Affiliations:** 1 Institute and Policlinic for Occupational and Social Medicine, Faculty of Medicine, Technische Universität Dresden, Fetscherstr. 74, D-01307 Dresden, Germany; 2 Institute of Social Medicine and Prevention, University Medicine Greifswald, Walther-Rathenau-Str. 48, D-17475 Greifswald, Germany; 3 German Centre for Cardiovascular Research (DZHK), Partner site Greifswald, Fleischmannstr. 42-44, D-17475 Greifswald, Germany; 4 Institute for Medical Psychology, University Medicine Greifswald, Walther-Rathenau-Str. 48, D-17475 Greifswald, Germany

## Abstract

**Background:**

The population impact of alcohol screening and brief intervention might be increased by approaching an entire population rather than individuals at high risk only. The aim is to present the protocol of the study “Testing a *pr*oactive expert system *int*ervention to prevent and to quit at-risk alcohol use” (PRINT) which tests the efficacy of a computer-based brief intervention (i) to elicit drinking reductions among persons with at-risk alcohol use and (ii) to prevent at-risk alcohol use among current low-risk drinkers.

**Methods/design:**

The PRINT study is a two-arm randomized controlled trial with a 12-month follow-up. A total of 1648 participants will be proactively recruited in the waiting area of a municipal registry office. All 18- to 64-year-old persons with past year alcohol use will be randomized to either the intervention group or the control group. Participants in the intervention group will receive computer-generated individualized feedback letters at baseline, month 3, and month 6. Participants in the control group will receive assessment only. The primary outcome is the change in the number of drinks per day from baseline to month 12.

**Discussion:**

We expect to provide a computer-based brief alcohol intervention that is appropriate for a wide range of people with alcohol use regardless of their initial alcohol-risk level. The intervention might have the potential to decrease alcohol use and alcohol-related problems on a population level at low costs.

**Trial registration:**

German Clinical Trials Register: DRKS00014274 (date of registration: 2018/03/12).

## Background

The World Health Organization calls for a 10% relative reduction of at-risk alcohol use by 2025 [1]. Screening and brief alcohol intervention (BAI) is a powerful tool to reduce alcohol use in entire populations [2], if delivered proactively. That is, each person of the target population is individually contacted by the intervention provider and offered BAI. Proactive outreach increases the probability that a large and representative part of the target population receives BAI [3].

Proactive recruitment is likely to result in considerable cost per participant. However, the intervention itself may be of low costs. An expert system software may automatically provide individualized feedback based on a person’s assessment data [4]. Expert system interventions have been found to be a cost-saving and efficacious alternative to in-person counseling among persons with at-risk alcohol use [5, 6].

About one quarter of the adult general population in Germany reports at-risk alcohol use [7]. There is a gap in the literature on potential BAI effects among the remaining 75%. As a substantial part of the BAI cost is produced by proactively contacting entire populations for alcohol screening, the cost-efficacy and the public health impact of BAI could be increased by providing more persons of the population with an appropriate intervention.

The majority of cases of alcohol-related diseases occur among the lesser-drinking majority of the population and not among the small proportion of particularly heavy drinkers [8]. A marginal drinking reduction among the high number of persons with low-risk alcohol use or even the maintenance of their low-risk drinking can have large effects on the population level. Furthermore, in particular moderate drinking patterns have been found to be subject to considerable short-term variations over time [9], which might increase the risk of excluding at-risk drinkers from BAI. For high risk groups of alcohol users, the evidence on (the lack of) BAI efficacy is inconclusive as most studies excluded people with particularly heavy alcohol use or dependence [10]. As pointed out by Heather [11], other factors than alcohol problem severity may be more important determinants of response to BAI, e.g., motivation to change. Also, intervention characteristics such as repeated contact and consistency of theory-based delivery might support particularly heavy drinkers in dealing with their alcohol problems. There is some promising evidence that BAI can be efficacious in the populations as a whole [12], especially in student populations [13–15].

The aim is to present the protocol of the study “Testing a *pr*oactive expert system *int*ervention to prevent and to quit at-risk alcohol use” (PRINT) which tests the efficacy of an expert system BAI among persons with alcohol use independent of whether or not the persons drink at risk or are particularly heavy drinkers. The intervention is expected to result in (i) drinking reductions among persons with at-risk alcohol use and (ii) prevention of at-risk alcohol use among current low-risk drinkers.

## Methods/design

### Study design

**Fig. 1:**
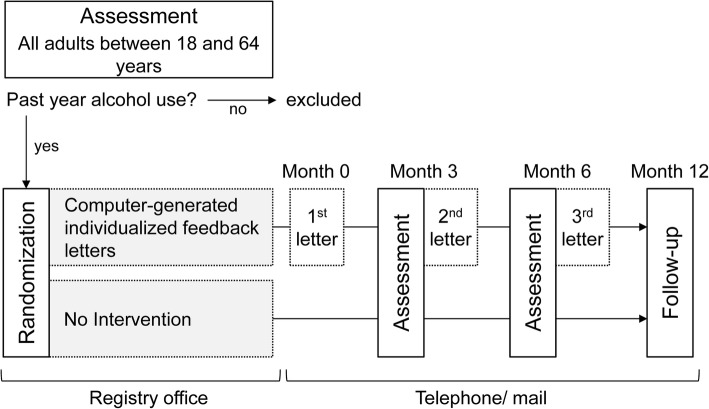
Study design

The PRINT study is a two-arm randomized controlled trial. Adults at age 18 to 64 years with past year alcohol use will be randomized to either the intervention group receiving computer-generated individualized feedback letters or the “assessment only” control group (Fig. 1). A 12-month follow-up will be conducted. The ethics committee of the University Medicine Greifswald, Germany, has approved the study (BB 147/15). The study is registered at the German Clinical Trials Register (DRKS00014274, date of registration: 2018/03/12).

### Study sample

#### Recruitment

Participants will be recruited proactively at the municipal registry office in Greifswald, Mecklenburg-West Pomerania, Germany. The registry office is the public authority for registration, passport and vehicle admission issues in Germany.

During opening hours, all 18- to 64-year-old registry office clients appearing in the waiting area will be approached by study assistants and asked to respond to questions about health risk behaviors provided by tablet computer. Those who agree will receive a brief introduction into the handling of the self-administered questionnaire. Persons cognitively or physically incapable, persons with insufficient language or reading skills, persons already approached during an earlier visit, escorting persons, and persons employed at the conducting research institute will be excluded.

Persons who report alcohol use in the previous twelve months are eligible and will be asked to participate in the PRINT trial. A study assistant will explain the purpose of the trial and the procedures involved. Participants will receive a study information sheet including study contact data. All persons need to provide written informed consent prior to participation in the PRINT trial. Those who report having no telephone or no permanent address will be excluded from trial participation. PRINT participants will receive a voucher of 5 €.

#### Sample size calculation

The primary outcome variable (number of drinks per day, see measures subsection) is expected to follow a negative binomial distribution. For a) μ_control_ = 10 drinks per week [16] and μ_intervention_ = 8.5 drinks per week [5] (15% intervention efficacy), b) a dispersion parameter of 1.0, c) 80% power, and d) 5% significance level (two-sided), a sample size of 659 per group are required [17]. Considering a 20% drop out [3, 18], a sample size of 824 per group is required.

### Measures

Baseline data will be collected using self-administered questionnaires provided on tablet computers. Three-, 6-, and 12-month assessments will be conducted via computer-assisted telephone interviews. All measures used in this study can be found in Table 1.

**Table 1:** Measures

Measures	Mo. 0	Mo. 3	Mo. 6	Mo. 12
Alcohol use				
12-month alcohol abstinence, 1 item	X			
Alcohol Use Disorders Identification Test [20], 10 items^a^	X	X	X	X
Alcohol use frequency and quantity past 30 days [3], 4 items	X	X	X	X
Drinks consumed on each day in the past week, 7 items	X	X	X	X
TTM constructs				
Stage of change, 4-item staging algorithm [3]	X	X_IG_	X_IG_	X
Alcohol Abstinence Self-Efficacy, 8-item short form [25]	X_IG_	X_IG_	X_IG_	
Alcohol Decisional Balance Scale, 10-item short form [25]	X_IG_	X_IG_	X_IG_	
Processes Of Change questionnaire, 20-item short form [29]	X_IG_	X_IG_	X_IG_	
Health and behavioral health risk factors				
Self-reported health, 1 item [23]	X	X	X	X
5-item Mental Health Inventory [22]	X	X	X	X
Tobacco use, 5 items	X	X	X	X
Fruit and vegetable intake, 1 item	X			X
Moderate to vigorous physical activity, 2 items	X			X
Height and weight, 2 items	X			X
Socio-demographics				
Sex, age, pregnancy, marital status, employment status	X	X	X	X
Years of school education, professional qualification	X			

Notes: Mo. month, X_IG_ intervention group only. ^a^item 3 modified: “How often do you have 4 [for women]/ 5 [for men] or more drinks on one occasion?”

#### Primary outcome

Primary outcome variable is the change in the number of drinks per day from baseline to month 12 determined by a quantity-frequency product based on two questions as used elsewhere [5]: (1) “In the past 30 days, how often did you have an alcoholic drink?” (never/ once/ 2–4 times/ 2–3 times per week/ 4 or more times per week) and (2) “In the past 30 days, how many drinks did you typically have on a drinking day?”. A drink is defined as 0.25–0.3 l beer, 0.1–0.15 l wine/ sparkling wine, or 4 cl spirits.

#### Secondary outcomes

Secondary outcome variables are changes in at-risk alcohol use, heavy drinking days in the past week, alcohol use problem severity, motivation to change, tobacco use, mental health, and self-reported health from baseline to month 12. At-risk alcohol use is determined using the Alcohol Use Disorders Identification Test-Consumption (AUDIT-C) [19] with a gender-specific version of the third item (“How often do you have 4 [*for women*]/ 5 [*for men*] or more drinks on one occasion?”) and a score of 4/5 or more for women/ men. The number of heavy drinking days in the past week is determined by asking “How many drinks did you have on each single day during the past seven days, starting with yesterday?” and summing the days with 4 [*for women*]/ 5 [*for men*] or more drinks. Alcohol use problem severity is assessed by the total AUDIT score [20]. Motivational stage of change (precontemplation/ contemplation/ preparation/ action) is assessed by a four-item staging algorithm described elsewhere [3]. If participants identified themselves as current smokers, their tobacco use is assessed by a frequency-quantity product based on two questions: (1) “On how many days per month do you smoke?” and (2) “How many cigarettes/ cigarillos/ cigars/ pipes do you typically smoke on a smoking day?”. Mental health is assessed by the five-item mental health inventory (MHI-5) [21, 22], self-rated health is assessed using the question “Would you say your health in general is: excellent/ very good/ good/ fair/ poor?” [23].

#### Variables required for the generation of individualized feedback

In addition to the motivational stage of change, the following constructs of the transtheoretical model of intentional behavior change (TTM) [24] are assessed: Self-efficacy is assessed by the eight-item short form of the Alcohol Abstinence Self-Efficacy scale (AASE) [25, 26], decisional balance by the ten-item short form of the Alcohol Decisional Balance Scale (ADBS) [25, 27], and processes of change are assessed by the 20-item short form of the Processes Of Change questionnaire (POC-20) [28, 29]. To comply with the target behavior of the intervention [30], the target element of the AASE and ADBS is changed from “abstaining from alcohol”/ “changing alcohol use” to “adhering to the low-risk drinking limits”. Low-risk drinking was defined as not exceeding the weekly limits of 7 alcohol drinks for women and 14 drinks for men and not exceeding the single occasion drinking limits of 3 drinks for women and 4 drinks for men [31].

Three alcohol-related risk levels were determined through the AUDIT [20]: AUDIT-C scores of 4/ 5 or more for women/ men and AUDIT scores below 20 indicate *at-risk alcohol use.* AUDIT scores of 20 or above indicate *more severe alcohol problems*. AUDIT-C scores below 4/ 5 for women/ men indicate *low-risk alcohol use*.

#### Other variables

Socio-demographic variables include sex, age, years of school education, professional qualification, partnership, employment status, and pregnancy. Behavior-related variables include self-reported weight and height, fruit and vegetable intake, and moderate-to-vigorous physical activity.

### Study groups and assignment procedure

#### Randomization

Participants will be assigned by tablet computer using a random generator to either the intervention group or the control group. The study staff will not be involved in group assignment.

#### Intervention group

Participants in the intervention group will receive computer-generated individualized feedback letters at baseline, month 3, and month 6. The feedback will be matched to (i) the motivational stage of change in accordance with the TTM [24] and the alcohol use problem severity. For persons with *at-risk alcohol use*, the 3–4-page letter includes the definition of low-risk drinking [31] as well as feedback on weekly alcohol use, heavy episodic drinking (HED), and TTM constructs in comparison to persons of the same sex, age, or stage of change. The letter refers to pages in a stage-matched manual about low-risk drinking [32]. Previous versions of the intervention for persons with at-risk alcohol use have been applied elsewhere and found to reduce alcohol use [5, 18]. Persons with *more severe alcohol use* additionally receive feedback on their perceived alcohol use disorder symptoms. The letter refers to pages in a self-help manual with a focus on problematic alcohol use and alcohol treatment [33]. For persons with *low-risk alcohol use*, the 2-page letter includes reinforcement of drinking within low-risk limits, the information that alcohol use can produce problems even within these limits (i.e., “low risk” is not “no risk”), and comparative feedback regarding weekly alcohol use and HED.

**Fig. 2:**
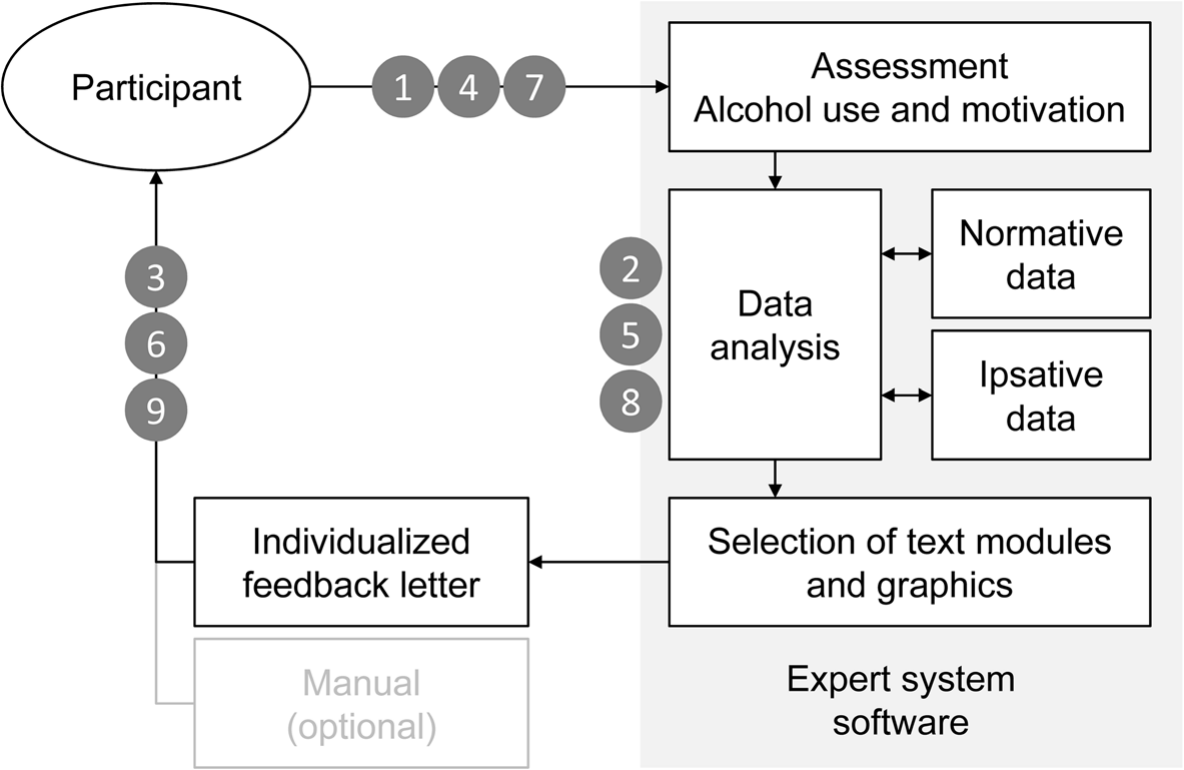
Expert system intervention according to Bischof et al. [4]: steps 1 to 9

As shown in Fig. 2, the intervention procedure in this study includes nine steps. *Step 1*: At baseline, participants respond to questions on alcohol use and TTM constructs provided by tablet computer in the waiting area of the registry office. *Step 2*: The expert system software analyzes data in comparison to general population data, selects supportive text modules and graphics, and generates a normative feedback letter. *Step 3*: The normative feedback letter is sent to the participants by mail. *Step 4*: Three months after baseline, computer-assisted telephone interviews including questions on alcohol use and TTM constructs are conducted. *Step 5*: The expert system software generates an ipsative feedback letter that include feedback on changes in alcohol use and TTM constructs (if appropriate) from baseline to month 3. *Step 6*: The ipsative feedback letter is sent to the participants by mail. *Step 7*: Six months after baseline, computer-assisted telephone interviews including questions on alcohol use and TTM constructs are conducted. *Step 8*: The expert system software generates a second ipsative feedback letter including feedback on changes in alcohol use and TTM constructs (if appropriate) from month 3 to 6. If persons were not reached at month 3, the letter would include feedback concerning changes from baseline to month 6. *Step 9*: The second ipsative feedback letter is sent to the participants by mail.

#### Control group

Participants in the control group will receive assessment at baseline, month 3, and month 6, including questions concerning alcohol use, health-related variables, and socio-demographics (see Table 1 for more details). Except of the stages of change, the control group assessment will not include TTM measures.

### Follow-up

Twelve-month follow-up assessments will be conducted via computer assisted telephone interviews. Assessors will be blinded to study group allocation. If 10 or more contact attempts fail, participants will receive an according questionnaire by mail or e-mail, with up to three reminders. Prior to 12-month follow-up assessment, all baseline participants will receive a voucher of 5 € by mail.

### Blinding

As part of the study procedure, participants will be informed at recruitment that they will receive either assessment and individualized feedback or assessment only. Due to the nature of the intervention, it is not possible to blind study assistants during the active intervention phase. During participant recruitment, baseline assessment, and 12-month follow-up, study assistants will be blinded to group allocation.

### Data handling, storage, and monitoring

Data from recruitment procedure (e.g., number of people approached, excluded and refused assessments) and assessment data will be collected computerized (via tablet computers at baseline or computer-assisted telephone interviews later). Process data (e.g., number of contacts) will be automatically recorded. All contacts via telephone, mail or e-mail will be recorded and time stamped. Data will be stored and analyzed pseudonymized, i.e., personal data is stored separately from scientific data. Publications will not include personal data. Data will not be publicly available due to potential privacy restrictions. To comply with the statement given in the informed consent procedure, the use of the data is restricted to medical research purposes.

Recruitment and follow-up participation rates will be monitored. Feedback letters will be checked and interviewers will be supervised regularly. Informed consents, exclusion criteria, and data quality (e.g., range checks for data values) will be checked.

### Statistical analysis

Data will be analyzed using latent growth curve modeling and maximum likelihood (ML) estimation. ML produces accurate model parameter under a missing at random assumption [34] and maximizes statistical power by using all available data [35]. In a latent growth model, repeated measures of the outcome variable are treated as indicators of latent growth variables representing the outcome growth trajectory. The form of the growth trajectory will be determined by time scores defined in the measurement model of the latent growth factors. Rescaled likelihood ratio tests will be used to decide on the form and variance of the growth trajectory. Outcome results will be expressed as net changes defined as study group differences between baseline and month 12. For the primary outcome variable, the net change will be given in incidence rate ratios (and 95% confidence intervals) indicating study group differences in the percentage change in alcohol use per day between baseline and 12-month follow-up. Analyses will be adjusted for sex, age, years of school education, employment status, and variables that are associated with non-participation at months 3, 6, and 12.

## Discussion

So far, BAI studies were primarily focused on selected at-risk populations. The rationale of this study is to increase the population impact of available expert system interventions to prevent and quit at-risk alcohol use in a non-medical setting. The expected main advantages are 1) increased prevention effects (i.e., maintenance of low-risk drinking), 2) increased effects on at-risk alcohol use and alcohol-related problems on the population level, and 3) higher rates of trial participation and utilization of help because of reduced stigma related to alcohol use. Further, expert system interventions yield the great potential for broad dissemination in various settings at low costs.

## Abbreviations

AASE: Alcohol Abstinence Self-Efficacy scale
ADBS: Alcohol Decisional Balance Scale
AUDIT-C: Alcohol Use Disorders Identification Test-Consumption
BAI: Brief Alcohol Intervention
HED: Heavy Episodic Drinking
MHI-5: Five-item Mental Health Inventory
ML: Maximum Likelihood
POC-20: 20-item Processes Of Change questionnaire
TTM: Trans-Theoretical Model of intentional behavior change
